# The Influence of Diabetes, Glycemic Control, and Diabetes-Related Comorbidities on Pulmonary Tuberculosis

**DOI:** 10.1371/journal.pone.0121698

**Published:** 2015-03-30

**Authors:** Chen Yuan Chiang, Kuan Jen Bai, Hsien Ho Lin, Shun Tien Chien, Jen Jyh Lee, Donald A. Enarson, Ting-I Lee, Ming-Chih Yu

**Affiliations:** 1 International Union Against Tuberculosis and Lung Disease, Paris, France; 2 Division of Pulmonary Medicine, Department of Internal Medicine, Wan Fang Hospital, Taipei Medical University, Taipei, Taiwan; 3 Department of Internal Medicine, School of Medicine, College of Medicine, Taipei Medical University, Taipei, Taiwan; 4 School of Respiratory Therapy, College of Medicine, Taipei Medical University, Taipei, Taiwan; 5 Institute of Epidemiology and Preventive Medicine, National Taiwan University, Taipei, Taiwan; 6 Chest Hospital, Department of Health, Tainan County, Taiwan; 7 Department of Internal Medicine, Tzu Chi General Hospital and Tzu Chi University, Hualien, Taiwan; 8 Division of Endocrinology and Metabolism, Department of Internal Medicine, Wan Fang Hospital, Taipei Medical University, Taipei, Taiwan; 9 Department of General Medicine, School of Medicine, College of Medicine, Taipei Medical University, Taipei, Taiwan; Graduate School of Medicine, Osaka University, JAPAN

## Abstract

**Background:**

To assess the influence of diabetes mellitus (DM), glycemic control, and diabetes-related comorbidities on manifestations and outcome of treatment of pulmonary tuberculosis (TB).

**Methodology/Principal Findings:**

Culture positive pulmonary TB patients notified to health authorities in three hospitals in Taiwan from 2005–2010 were investigated. Glycemic control was assessed by glycated haemoglobin A1C (HbA1C) and diabetic patients were categorized into 3 groups: HbA1C<7%, HbA1C 7–9%, HbA1C>9%. 1,473 (705 with DM and 768 without DM) patients were enrolled. Of the 705 diabetic patients, 82 (11.6%) had pretreatment HbA1C<7%, 152 (21.6%) 7%–9%, 276 (39.2%) >9%, and 195 (27.7%) had no information of HbA1C. The proportions of patients with any symptom, cough, hemoptysis, tiredness and weight loss were all highest in diabetic patients with HbA1C>9%. In multivariate analysis adjusted for age, sex, smoking, and drug resistance, diabetic patients with HbA1C>9% (adjOR 3.55, 95% CI 2.40–5.25) and HbA1C 7–9% (adjOR 1.62, 95% CI 1.07–2.44) were significantly more likely to be smear positive as compared with non-diabetic patients, but not those with HbA1C<7% (adjOR 1.16, 95% CI 0.70–1.92). The influence of DM on outcome of TB treatment was not proportionately related to HbA1C, but mainly mediated through diabetes-related comorbidities. Patients with diabetes-related comorbidities had an increased risk of unfavorable outcome (adjOR 3.38, 95% CI 2.19–5.22, p<0.001) and one year mortality (adjOR 2.80, 95% CI 1.89–4.16). However, diabetes was not associated with amplification of resistance to isoniazid (p = 0.363) or to rifampicin (p = 0.344).

**Conclusions/Significance:**

Poor glycemic control is associated with poor TB treatment outcome and improved glycemic control may reduce the influence of diabetes on TB.

## Introduction

The International Diabetes Federation has estimated that the number of people living with diabetes mellitus (DM) worldwide has been increasing and will rise to 592 million by 2035.[1] Several studies have shown that DM is associated with an increased risk of tuberculosis (TB).[2] A recent meta-analysis reported that the relative risk of TB in diabetic patients was 3.11 (95% CI 2.27–4.26) as compared with individuals without DM in cohort studies.[3] The potential impact of a rising epidemic of DM on TB has been raised in several articles.[4–6] To address the dual challenge of DM and TB, the World Health Organization and the International Union Against Tuberculosis and Lung Disease have recently published a collaborative framework for care and control of TB and DM.[7]

The influence of DM on clinical manifestations and outcome of treatment of pulmonary TB has previously been reported. [8–13] However, the results reported by different researchers have not been consistent. Leung et al reported that the risk of TB in elderly diabetic patients was associated with glycemic control.[14] As clinical manifestations of pulmonary TB are likely related to immune status and hyperglycemia is associated with changes of immune response, we hypothesized that the influence of DM on clinical manifestations of pulmonary TB is related to glycemic control.[15, 16] As complications of chronic hyperglycemia may have an impact on outcome of TB treatment, we further hypothesized that this effect is mediated through diabetes-related comorbidities. We report the results of a study on the influence of DM, glycemic control, and diabetes-related comorbidities on pulmonary TB.

## Materials and Methods

Setting: Three teaching hospitals located in each of Northern, Eastern and Southern Taiwan. A list of all TB patients notified to health authorities from 2005–2010 who were managed by these three hospitals was obtained from the national TB registry at Taiwan CDC.

Objectives: to investigate whether the influence of DM on clinical manifestations of pulmonary TB is related to glycemic control and whether the impact of chronic hyperglycemia on outcome of TB treatment is mediated through diabetes-related comorbidities.

Patient material and definitions: Notification data and patients’ medical records were reviewed to identify culture positive pulmonary TB patients. Patients who 1) were treated with insulin or diabetes-specific hypoglycemic agents, 2) had been assigned a diabetes-related International Classification of Diseases 9th Revision (ICD-9) code during admission, 3) had been assigned a diabetes-related ICD-9 code 2 or more times on an outpatient visit, or 4) had a history of diabetes, were considered as probably having diabetes and were assessed. Patients who were found to have transient hyperglycemia at the initiation of anti-TB treatment were excluded. A non-diabetic culture positive pulmonary TB patient who had never been documented to have 1) glycated haemoglobin A1C (HbA1C) ≧6.5%, or 2) fasting plasma glucose≧126 mg/dl, or 3) post-prandial plasma glucose≧200 mg/dl, or 4) random plasma glucose≧200 mg/dl and was notified to health authority immediately prior to each probable diabetic TB patient was identified as a comparison. Culture positive TB patients who started anti-TB treatment within 3 months of sputum collection were included in this study.

Study procedures: Data were collected from medical charts using a structured questionnaire. Data collected included age, sex, pretreatment smear (negative vs positive, and positivity grade, scanty, 1+, 2+, 3+ 4+), type of TB case (new vs retreatment), smoking status (ever vs never), comorbidities, culture at 2 months, and outcome of treatment. Glycemic control was assessed by HbA1C measured within 3 months of the initiation of TB treatment; diabetic patients were categorized into 3 groups: HbA1C<7%, HbA1C 7–9%, HbA1C>9%. Comorbidity was categorized as 1) non-diabetes-related comorbidity, including cancer, pneumoconiosis, cirrhosis, and HIV, and 2) diabetes-related comorbidity, including chronic renal, cardiovascular, and cerebrovascular disease. Pretreatment drug susceptibility testing of isoniazid (H), rifampicin (R), ethambutol and streptomycin were collected and patients were classified as 1) susceptible, 2) any resistance to H but not R (HrRs), 3) resistance to at least both H and R (HrRr), and 4) other resistance patterns.

Outcome of TB treatment was categorized as treatment success (documented sputum culture conversion and remained culture negative till completion of a treatment course), failed (sputum culture positive at 5 months of treatment or later), lost-to-follow-up (interruption of treatment for 2 consecutive months or lack of outcome assessment) and died (died of any cause during TB treatment).

### Sample size

The required sample size was estimated using the following assumptions:

1. The proportion of diabetic patients with proper glycemic control is 50%
2. Odds ratio of importance regarding manifestations and outcome of tuberculosis is at least 2.5

Applying a 95% 2-sided confidence interval and 80% power, the number of participants required to satisfactorily address the hypothesis, given the assumptions, was 336. We estimated that the number of diabetic TB patients notified to health authorities from 2005–2010 who were managed at the three hospitals was much higher than 336 and decided to include all diabetic TB cases to account for the uncertainty of assumptions, the effects of other variables as well as those in whom information collected was not complete.

### Data entry and analysis

To ensure accuracy of data entry, the data set was double entered and validated using EpiData Entry 3.1 (EpiData Association, Odense, Denmark). Discrepant records were checked and corrected against the original data on the questionnaires. STATA Version 12 (StataCorp LP, College Station, Texas, USA) was used for statistical analysis. Categorical data were analyzed by Pearson Chi-square test. Logistic regression models were constructed for outcome variables with 2 categories (symptoms, pretreatment smear, 2-month culture, and treatment outcome) and multinomial logistic regression for that with 3 categories or more (smear positivity grades). In treatment outcome analysis, outcome was further dichotomized into successful (treatment success) and unfavorable (died, failed, loss-to-follow-up). Two multivariate logistic regression models were constructed. The first model assessed the association of diabetes and unfavorable outcome adjusted for age, sex, smear, type of case, smoking, drug resistance and non-diabetes-related comorbidity. The second model included diabetes-related comorbidity to assess whether the effect of diabetes on outcome of TB treatment was mediated through diabetes-related comorbidity.

To assess risk factors associated with one year mortality, all patients were followed up from the initiation of anti-TB treatment until death or last contact. Cases with a follow-up of ⩾1 year were censored at one year. Kaplan-Meier survival estimates and logrank test were used to evaluate factors associated with one year mortality. All relevant variables were entered into a multivariate Cox proportional hazards model, and a final fitted model was determined by backward elimination using the likelihood ratio test. The final model was checked by diagnostics including link test, graphical methods and residual analysis.

A p-value less than 0.05 was considered statistically significant.

### Ethics

This study was approved by the Joint Institute Review Board of Taipei Medical University. Written informed consent by participants for their clinical records to be used in this study was waived. Patient information was anonymized and de-identified prior to analysis.

## Results

A total of 1,594 (797 with probable DM and 797 without DM) culture positive pulmonary TB patients were assessed. Of the 797 patients with probable DM, 717 were confirmed to have DM. Of the 1,514 patients (717 with DM and 797 without DM), 1,473 (705 with DM and 768 without DM) patients started anti-TB treatment within 3 months of sputum collection and were included in this study.

**Fig 1:**
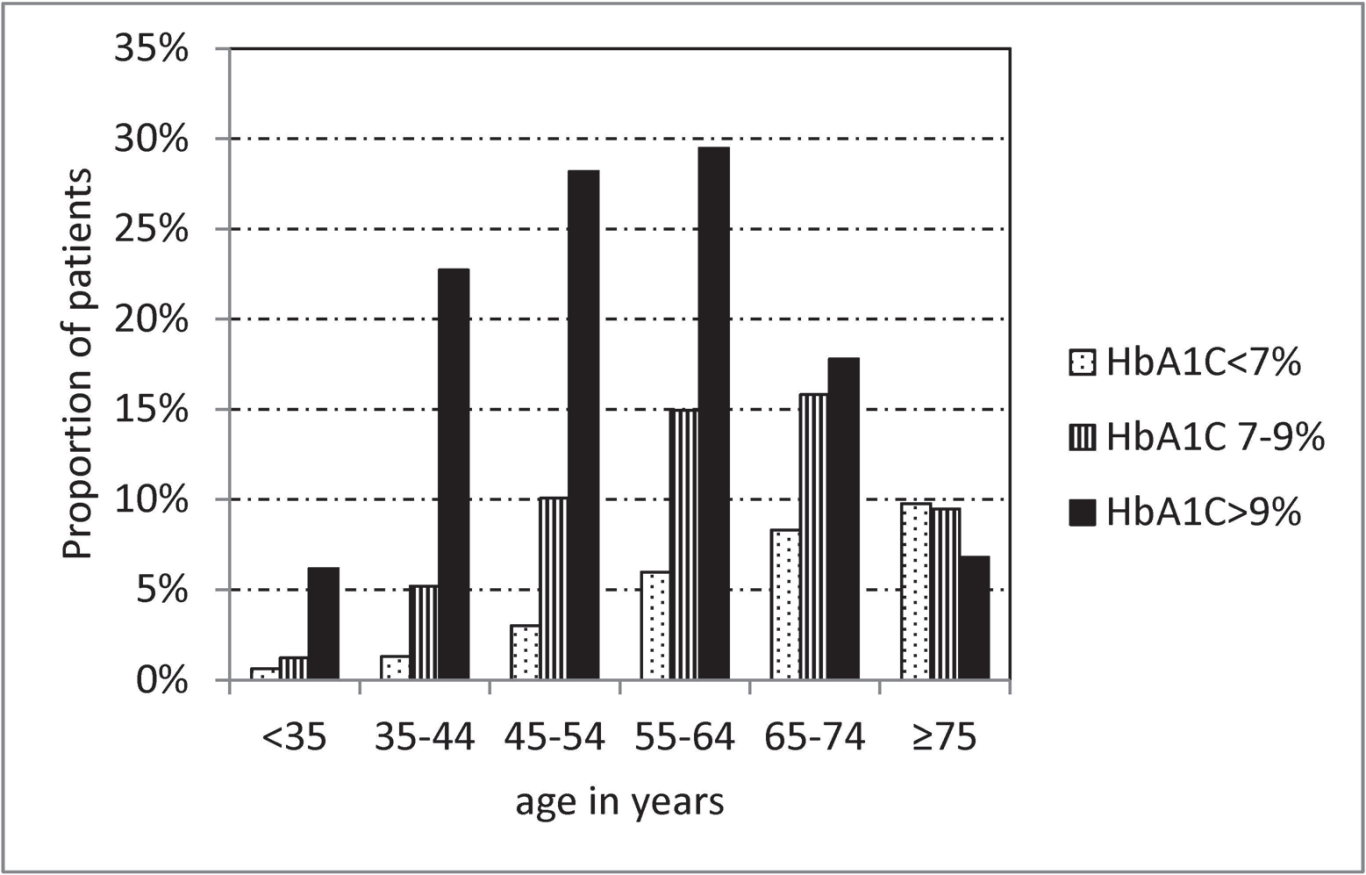
Proportion of patients with HbA1C<7%, HbA1C 7–9%, HbA1C>7% by age groups of consecutive culture positive pulmonary tuberculosis patients with diabetes mellitus who had results of pretreatment HbA1C treated in three referral hospitals in Taiwan, 2005–2010.

Of the 1,473 culture positive TB patients, 108 (7.3%) had non-diabetes-related comorbidities (cancer = 66 (4.5%), pneumoconiosis = 7 (0.5%), cirrhosis = 38 (2.6%), and human immunodeficiency virus (HIV) infection = 5 (0.3%)), and 128 (8.7%) had diabetes-related comorbidities (cardiovascular diseases = 65(4.4%), cerebrovascular diseases = 35 (2.4%), chronic renal disease = 95 (6.5%)) (Table 1). Of the 705 TB patients with DM, 574 (81.4%) were diagnosed with DM prior to the diagnosis of TB; 82 (11.6%) had pre-treatment HbA1C<7%, 152 (21.6%) 7%-9%, 276 (39.2%) >9%, and 195 (27.7%) had no information of HbA1C. Fig. 1 shows that HbA1C was significantly associated with age (p<0.001); the proportion of patients with HbA1C>9% was >20% in patients aged 35–64 years.

**Table 1:** Characteristics of consecutive culture positive pulmonary tuberculosis patients with diabetes mellitus treated in three referral hospitals in Taiwan, 2005–2010 compared with a selected group of tuberculosis patients treated in the same hospitals but without diabetes mellitus.

	Total		Diabetes		Non-Diabetes		P value
	N	col %	N	col %	N	col %	
Total	1,473	100.0	705	100.0	768	100.0	
Sex							0.004
Male	1,094	74.3	548	77.7	546	71.1	
Female	379	25.7	157	22.3	222	28.9	
Age group (years)							<0.001
<35	162	11.0	14	2.0	148	19.3	
35–44	154	10.5	63	8.9	91	11.9	
45–54	298	20.2	166	23.6	132	17.2	
55–64	268	18.2	181	25.7	87	11.3	
65–74	253	17.2	137	19.4	116	15.1	
≧75	338	23.0	144	20.4	194	25.3	
Type of TB							0.966
New	1,273	86.4	609	86.4	664	86.5	
Previously treated	200	13.6	96	13.6	104	13.5	
Smear							<0.001
Negative	480	32.6	177	25.1	303	39.5	
Positive	862	58.5	469	66.5	393	51.2	
Scanty	17	1.2	14	2.0	3	0.4	
1+	174	11.8	88	12.5	86	11.2	
2+	161	10.9	88	12.5	73	9.5	
3+	192	13.0	102	14.5	90	11.7	
4+	318	21.6	177	25.1	141	18.4	
Unknown	131	8.9	59	8.4	72	9.4	
Smoking							0.001
Never	759	51.5	330	46.8	429	55.9	
Ever	688	46.7	365	51.8	323	42.1	
Unknown	26	1.8	10	1.4	16	2.1	
Drug resistance*							0.567
Susceptible	1,086	73.7	522	74.0	564	73.4	
HrRs	113	7.7	58	8.2	55	7.2	
HrRr	60	4.1	32	4.5	28	3.7	
Other	48	3.3	21	3.0	27	3.5	
Not done	166	11.3	72	10.2	94	12.2	
Non-DM-Com^†^							0.008
Yes	108	7.3	65	9.2	43	5.6	
No	1365	92.7	640	90.8	725	94.4	
DM-Com^‡^							<0.001
Yes	128	8.7	114	16.2	14	1.8	
No	1345	91.3	591	83.8	754	98.2	

* Susceptible, susceptible to isoniazid, rifampicin, ethambutol, and streptomycin; HrRs, any resistance to isoniazid but not resistance to rifampicin; HrRr, resistance to at least both isoniazid and rifampicin; other, other resistant patterns.

† Non-DM-Com, non-diabetes-related comorbidity, including cancer, pneumoconiosis, cirrhosis and HIV.

‡ DM-Com, diabetes-related comorbidity, including chronic renal, cardiovascular, and cerebrovascular diseases.

**Fig 2:**
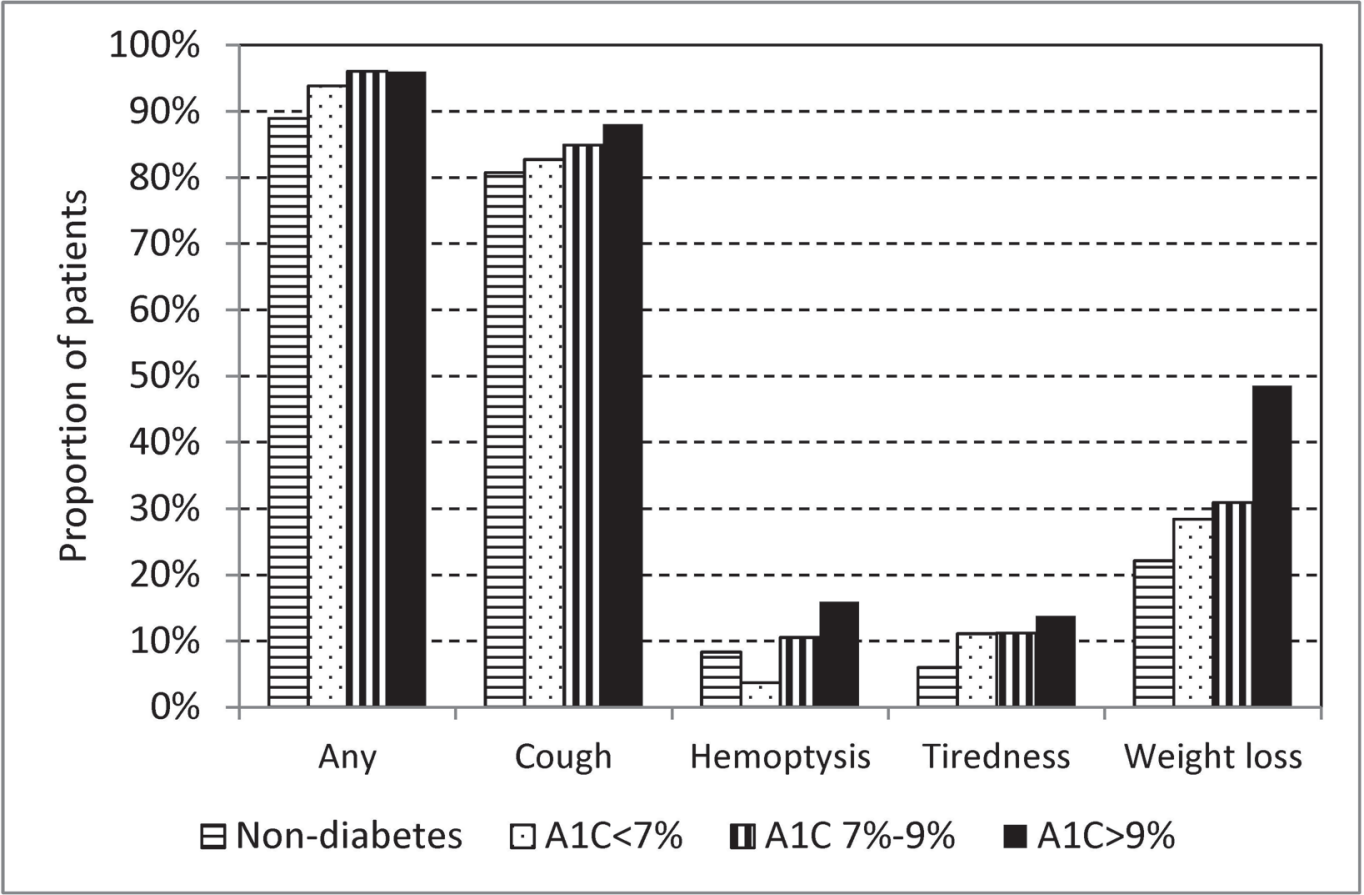
Proportion of patients with any symptom, cough, hemoptysis, and weight loss of consecutive culture positive pulmonary tuberculosis patients with diabetes mellitus treated in three referral hospitals in Taiwan, 2005–2010 compared with a selected group of tuberculosis patients treated in the same hospitals but without diabetes mellitus, and influence of HbA1C (AIC) level.

Of the 1,473 culture positive TB patients, 1,356 (92%) had any symptom (95.6% in diabetes, 88.9% in non-diabetes, p<0.001), 1,228 (83.4%) cough (86.4% in diabetes, 80.7% in non-diabetes, p = 0.004), 151 (10.3%) hemoptysis (12.4% in diabetes, 8.3% in non-diabetes, p = 0.011), 126 (8.7%) tiredness (11.4% in diabetes, 6.0% in non-diabetes, p<0.001), 417 (28.3%) weight loss (35.1% in diabetes, 22.1% in non-diabetes, p<0.001). In a multivariate logistic regression model adjusted for age, sex, type of case and smoking, diabetic patients were significantly more likely to have any symptom (adjusted odds ratio (adjOR) 2.38, 95% confidence interval (CI) 1.52–3.73), cough (adjOR 1.35, 95% CI 1.01–1.84), hemoptysis (adjOR 1.85, 95% CI 1.26–2.72), tiredness (adjOR 1.70, 95% CI 1.14–2.52), and weight loss (adjOR 1.75, 95% CI 1.37–2.24) as compared with non-diabetic patients. The presence of symptoms in diabetes was influenced by HbA1C. Fig. 2 shows that the proportions of patients with any symptom, cough, hemoptysis, tiredness and weight loss were all highest in diabetic patients with HbA1C>9% as compared with other groups. Diabetic patients with HbA1C>9% were significantly more likely to have any symptom, hemoptysis, tiredness and weight loss in multivariate logistic regression models adjusted for age, sex, type of case and smoking as compared with non-diabetic patients (data not shown).

Of the 1,473 culture positive TB patients, 862 (58.5%) were smear positive (Table 1). Diabetes (p<0.001), HbA1C (p<0.001), age (p<0.001), smoking (p<0.001), drug resistance (p<0.001) were significantly associated with positive smear but not sex (p = 0.161), type of case (p = 0.974), non-diabetes-related comorbidity (p = 0.974), and diabetes-related comorbidity (p = 0.673). The proportion of patients without smear results did not differ between diabetes and non-diabetes (8.4% vs 9.4%, p = 0.498). Diabetic patients were significantly more likely to be smear positive as compared with non-diabetic patients (adjOR 1.99, 95% CI 1.55–2.56) and that the association between diabetes and positive smear was significantly influenced by HbA1C. In multivariate analysis adjusted for age, sex, smoking, and drug resistance, diabetic patients with HbA1C>9% (adjOR 3.55, 95% CI 2.40–5.25) and those HbA1C 7–9% (adjOR 1.62, 95% CI 1.07–2.44) were significantly more likely to be smear positive as compared with non-diabetic patients, but not those with HbA1C<7% (adjOR 1.16, 95% CI 0.70–1.92).

**Fig 3:**
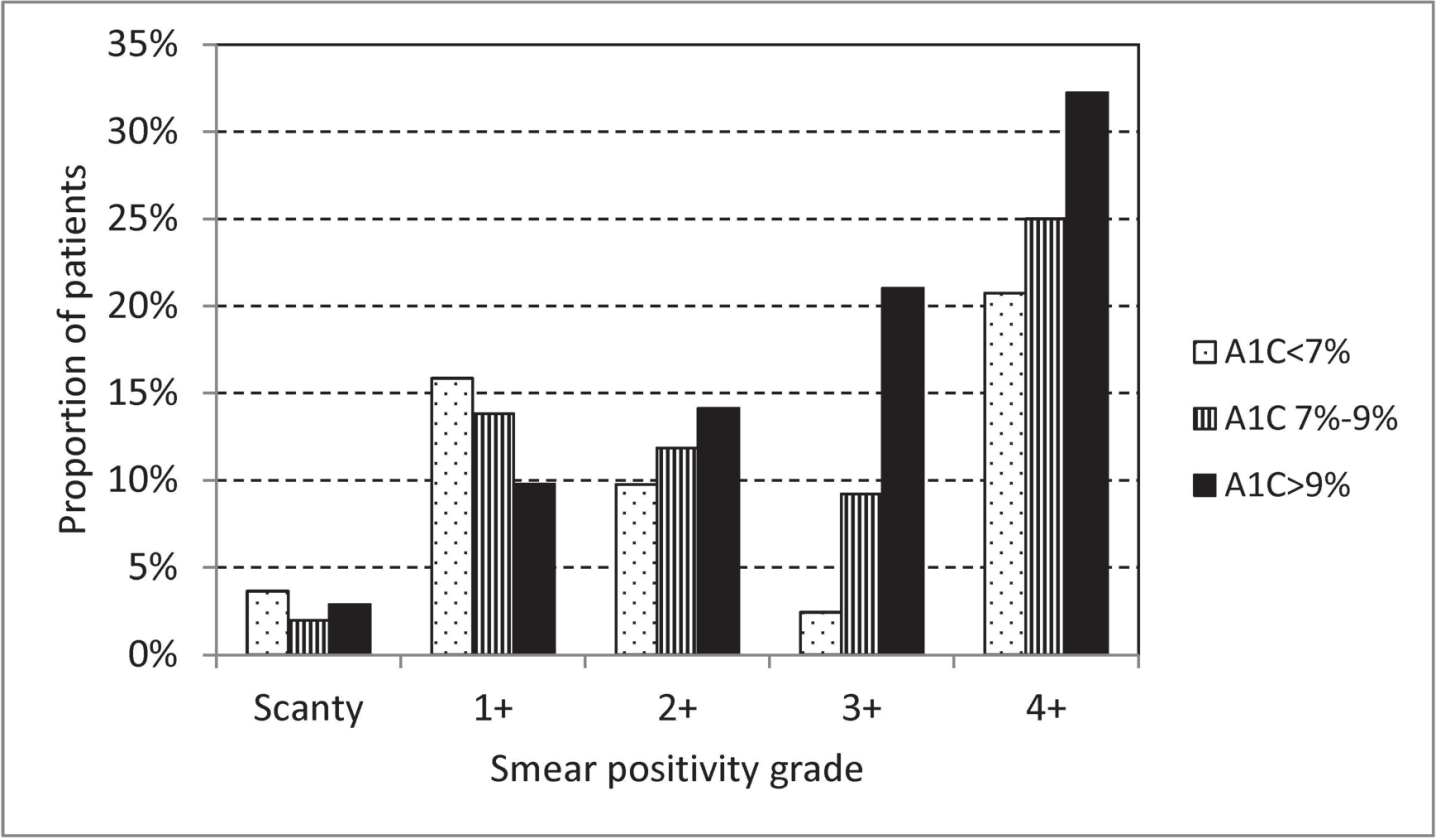
Association of pre-treatment smear positivity grades in smear positive pulmonary tuberculosis patients, by HbA1C (A1C) among consecutive culture positive pulmonary tuberculosis patients with diabetes mellitus who had results of pretreatment HbA1C treated in three referral hospitals in Taiwan, 2005–2010.

The proportion of patients with a high smear positivity grade (3+ or 4+) was significantly higher among diabetic patients than non-diabetic patients (43.2% vs 33.2%, p<0.001). In multinomial logistic regression, the adjusted relative risk of a high smear positivity grade (3+ or 4+) for diabetic patients relative to non-diabetic patients was 1.97 (95% CI 1.46–2.66) (smear negative as base for comparison, adjusted for age, sex, smoking and drug resistance). The association between diabetes and high smear positivity grade was significantly influenced by the level of HbA1C (Fig. 3). In multinomial logistic regression, the adjusted relative risk of a high smear positivity grade (3+ or 4+) was 4.03 (95% CI 2.66–6.10) for patients with HbA1C >9%, 1.55 (95% CI 0.98–2.45) for patients with HbA1C 7%-9%, and 0.95 (95% CI 0.51–1.77) for patients with HbA1C <7%, relative to non-diabetic patients (smear negative as base for comparison, adjusted for age, sex, smoking and drug resistance).

At 2 months of anti-TB treatment, 671 (45.9%) patients had culture conversion, 202 (13.7%) remained culture positive for *M*. *tuberculosis*, 600 (40.7%) had no data of sputum culture at 2 months. Diabetes (p = 0.002), HbA1C (p = 0.002), sex (p = 0.008), age (p = 0.001), smear (p<0.001), smoking (p<0.001), and drug resistance (p = 0.023) were significantly associated with positive culture at 2 months but not type of case (p = 0.671), non-diabetes-related comorbidity (p = 0.079), or diabetes-related comorbidity (p = 0.079). The proportion TB patients without information of sputum culture at 2 months was not significantly different between diabetes and non-diabetes (38.3% vs 43.0% p = 0.068). Among patients with results of sputum culture at 2 months of treatment, diabetic patients were significantly more likely to be culture positive as compared with non-diabetic patients (adjOR 1.63, 95% CI 1.14–2.34), and that the association was significantly influenced by the level of HbA1C. In multivariate analysis adjusted for age, sex, smoking, and drug resistance, diabetic patients with HbA1C>9% were significantly more likely to be culture positive at 2 months (adjOR 1.65, 95% CI 1.05–2.58) as compared with non-diabetic patients, but not those with HbA1C<7% (adjOR 1.56, 95% CI 0.77–3.18), and those HbA1C 7–9% (adjOR 1.58, 95% CI 0.89–2.81).

Table 2 shows outcome of treatment of the 1,473 culture positive TB patients. Age (p<0.001), diabetes (p = 0.002), HbA1C (p<0.001), type of case (p = 0.009), drug resistance (p<0.001), diabetes-related comorbidity (p<0.001), and non-diabetes-related comorbidity (p<0.001) were significantly associated with outcome of treatment. Treatment success proportion in patients aged≧75 years was only 76.3%, due to 21.3% who died. Treatment success proportion in diabetic patients was 83.7%, significantly lower than 89.5% in non-diabetic patients. Treatment success proportion was inversely related to HbA1C (78.1% in diabetic patients with HbA1C<7%, 82.2% in HbA1C 7%-9%, and 88.4% in HbA1C>9%), mainly because of differential risk of death (18.3%, 13.8%, and 5.8%, respectively). Treatment success proportion in patients with non-diabetes-related comorbidities was 66.0%, significantly lower than 88.3% in those without. Similarly, treatment success proportion in patients with diabetes-related comorbidities was 63.3%, significantly lower than 88.9% in those without.

**Table 2:** Outcome of treatment of consecutive culture positive pulmonary tuberculosis patients with diabetes mellitus treated in three referral hospitals in Taiwan, 2005–2010 compared with a selected group of tuberculosis patients treated in the same hospitals but without diabetes mellitus.

	Success		Died		Lost		Failed		
	N	%	N	%	N	%	N	%	
Total	1,277	86.7	140	9.5	17	1.2	39	2.7	
Sex									0.183
Male	936	85.6	112	10.2	14	1.3	32	2.9	
Female	341	90.0	28.0	7.4	3	0.8	7.0	1.9	
Age (years)									<0.001
<44	302	95.6	5	1.6	2	0.6	7	2.2	
45–64	505	89.2	28	5.0	9	1.6	24	4.2	
65–74	212	83.8	35	13.8	1	0.4	5	2.0	
≧75	258	76.3	72	21.3	5	1.5	3	0.9	
Diabetes									0.002
No	687	89.5	61	7.9	3	0.4	17	2.2	
Yes	590	83.7	79	11.2	14	2.0	22	3.1	
A1C<7%	64	78.1	15	18.3	0	0.0	3	3.7	<0.001
A1C 7%-9%	125	82.2	21	13.8	2	1.3	4	2.6	
A1C>9%	244	88.4	16	5.8	7	2.5	9	3.3	
Unknown	157	80.5	27	13.9	5	2.6	6	3.1	
Smear									<0.001
Negative	431	89.8	39	8.1	3	0.6	7	1.5	
Positive	734	85.2	91	10.6	7	0.8	30	3.5	
Unknown	112	85.5	10	7.6	7	5.3	2	1.5	
Type of TB									0.009
New	1,118	87.8	113	8.9	13	1.0	29	2.3	
Retreatment	159	79.5	27	13.5	4	2.0	10	5.0	
Smoking									0.004
Never	675	88.9	66	8.7	8	1.1	10	1.3	
Ever	581	84.5	72	10.5	9	1.3	26	3.8	
Unknown	21	80.8	2	7.7	0	0	3	11.5	
Drug resistance*									<0.001
Susceptible	956	88.0	98	9.0	5	0.5	27	2.5	
HrRs	92	81.4	16	14.2	3	2.7	2	1.8	
HrRr	44	73.3	9	15.0	2	3.3	5	8.3	
Other	41	85.4	4	8.3	1	2.1	2	4.2	
Not done	144	86.8	13	7.8	6	3.6	3	1.8	
Non-DM-Com^†^									<0.001
No	1209	88.3	112	8.2	15	1.1	34	2.5	
Yes	68	66.0	28	27.2	2	1.9	5	4.9	
DM-Com^‡^									<0.001
No	1,196	88.9	99	7.4	14	1.0	36	2.7	
Yes	81	63.3	41	32.0	3	2.3	3	2.3	

* HrRs, any resistance to isoniazid but not resistance to rifampicin; HrRr, resistance to at least both isoniazid and rifampicin; other, other resistant pattern.

† Non-DM-Com, non-diabetes-related comorbidity, including cancer, pneumoconiosis, cirrhosis, and HIV.

‡ DM-Com, diabetes-related comorbidity, including chronic renal, cardiovascular, and cerebrovascular diseases.

In univariate analysis, diabetic patients were significantly less likely to have treatment success (OR 0.61, 95% CI 0.45–0.82), and had a significantly increased risk of death (OR 1.46, 95% CI 1.03–2.08), loss-to-follow-up (OR 5.17, 95% CI 1.48–18.05) but not failure (OR 1.42, 95% CI 0.75–2.70). When outcome was dichotomized into successful and unfavorable (death, loss and failed), both DM and diabetes-related comorbidities were significantly associated with unfavorable outcome. (Table 3) In a multivariate logistic regression model adjusted for age, sex, type of case, smear, smoking and drug resistance, diabetes is significantly associated with an increased risk of unfavorable outcome (adjOR 1.57, 95% CI 1.14–2.18, p = 0.006), as compared with non-diabetes. When diabetes-related comorbidity was included in the multivariate model, diabetes-related comorbidity was significantly associated with unfavorable outcome but the association between diabetes and unfavorable outcome was attenuated and no longer statistically significant (adjOR 1.09, 95% CI 0.76–1.56, p = 0.643). Patients with diabetes-related comorbidities had an increased risk of unfavorable outcome (adjOR 3.26, 95% CI 2.05–5.17, p<0.001), as compared with non-diabetes. (Table 3)

**Table 3:** Diabetes, glycemic control, and comorbidities and outcome of tuberculosis treatment in univariate and multivariate analysis of consecutive culture positive pulmonary tuberculosis patients with diabetes mellitus treated in three referral hospitals in Taiwan, 2005–2010 compared with a selected group of tuberculosis patients treated in the same hospitals but without diabetes mellitus.

	Unfavorable		Univariate		Multivariate Model 1		Multivariate Model 2	
	N	%	OR	95% CI	AdjOR	95% CI	AdjOR	95% CI
Diabetes								
No	81	10.6	1		1		1	
Yes	115	16.3	1.65	1.22–2.24	1.42	1.02–1.99	1.09	0.76–1.56
Sex								
Male	158	14.4	1.51	1.04–2.20				
Female	38	10.0	1					
Age (years)								
<44	14	4.4	1		1		1	
45–64	61	10.8	2.61	1.43–4.74	2.03	1.09–3.78	2. 05	1.10–3.83
65–74	41	16.2	4.17	2.22–7.85	3.97	2.06–7.64	3.60	1.86–6.98
≧75	80	23.7	6.69	3.70–12.09	7.61	4.10–14.1	6.48	3.46–12.11
Smear								
Negative	49	10.2	1		1		1	
Positive	128	14.9	1.53	1.08–2.18	1.87	1.27–2.74	1.95	1.32–2.89
Unknown	19	14.5	1.49	0.84–2.64	1.50	0.81–2.77	1.54	0.83–2.86
Smoking								
Never	84	11.1	1		1		1	
Ever	107	15.6	1.48	1.09–2.01	1.46	1.04–2.04	1.53	1.09–2.15
Unknown	5	19.2	1.91	0.70–5.21	2.49	0.86–7.19	1.91	0.64–5.70
Case type								
New	155	12.2	1		1		1	
Retreatment	41	20.5	1.86	1.27–2.73	1.70	1.12–2.60	1.76	1.15–2.69
Drug resistance*								
Susceptible	130	12.0	1		1		1	
HrRs	21	18.6	1.68	1.01–2.79	1.77	1.02–3.04	1.85	1.06–3.20
HrRr	16	26.7	2.67	1.47–4.88	3.35	1.71–6.56	3.45	1.74–6.81
Other	7	14.6	1.26	0.55–2.86	1.36	0.57–3.28	1.27	0.53–3.09
Not done	22	13.3	1.12	0.69–1.82	1.32	0.78–2.24	1.48	0.87–2.54
Non-DM-Com^†^								
No	160	11.7	1		1		1	
Yes	36	33.3	3.77	2.44–5.81	3.23	2.03–5.13	2.95	1.83–4.75
DM-Com^‡^								
No	149	11.1	1				1	
Yes	47	36.7	4.66	3.13–6.93			3.26	2.05–5.17

* HrRs, any resistance to isoniazid but not resistance to rifampicin; HrRr, resistance to at least both isoniazid and rifampicin; other, other resistance patterns.

† Non-DM-Com, non-diabetes-related comorbidity, including cancer, pneumoconiosis, cirrhosis and HIV.

‡ DM-Com, diabetes-related comorbidity, including chronic renal, cardiovascular, and cerebrovascular diseases.

A total of 130 patients died within one year of anti-TB treatment. Age (p<0.001), non-diabetes-related comorbidity (p<0.001) and diabetes-related comorbidity (p<0.001) were significantly associated with one year mortality but not diabetes (p = 0.249), sex (p = 0.183), smear (p = 0.179), type of case (p = 0.139), smoking (p = 0.629), and drug resistance (p = 0.544).Patients with diabetes-related comorbidities had a significantly higher risk of one year mortality (adjusted hazard ratio 2.80, 95% CI 1.89–4.16) than those without. (Table 4)

**Table 4:** Factors associated with one year mortality of consecutive culture positive pulmonary tuberculosis patients with diabetes mellitus treated in three referral hospitals in Taiwan, 2005–2010 compared with a selected group of tuberculosis patients treated in the same hospitals but without diabetes mellitus.

	Total N	Death N (%)	Adjusted Hazard Ratio	P value	95% confidence interval	
Age group (years)						
<65	882	28(3.2)	1			
65 or more	591	102(17.3)	5.06	<0.01	3.31	7.75
Non-DM-Com*						
No	1365	102(7.5)	1			
Yes	108	28(25.9)	2.81	<0.01	1.83	4. 23
DM-Com†						
No	1345	93(6.1)	1			
Yes	128	37(28.9)	2.80	<0.01	1.89	4.16

* Non-DM-Com, non-diabetes-related comorbidity, including cancer, pneumoconiosis, liver cirrhosis, and HIV.

† DM-Com, diabetes-related comorbidity, including chronic renal diseases, cardiovascular diseases, and cerebrovascular diseases.

Of the 1,473 patients, 17(1.2%) had amplification of resistance to H, 10 (1.4%) in diabetic patients and 7 (0.9%) in non-diabetic patients and 15(1.0%) had amplification of resistance to R, 9 (1.3%) in diabetic patients and 6 (0.8%) in non-diabetic patients. Diabetes was not associated with amplification of resistance to H (p = 0.363) or to R (p = 0.344).

## Discussion

Our study demonstrates that clinical manifestations of pulmonary TB in diabetic patients are related to pre-treatment HbA1C. The proportions of diabetic patients with symptoms and positive smear were all highest in patients with HbA1C>9%. Our study further demonstrates that the influence of DM on outcome of TB treatment was not proportionately related to pretreatment HbA1C, but mainly mediated through diabetes-related comorbidities. Patients with diabetes-related comorbidities had an increased risk of unfavorable outcome and one year mortality. However, diabetes was not associated with amplification of resistance to H or to R.

### Clinical manifestations of pulmonary TB

The influence of DM on clinical manifestations of pulmonary TB has previously been reported. Alisjahbana et al reported that diabetic TB patients had more symptoms, but not a higher frequency of positive sputum smears for acid-fast bacilli (AFB).[17] Wang et al reported that diabetic TB patients had higher frequencies of fever, haemoptysis, and positive AFB sputum smears.[18] Chang et al report that diabetics are more likely to be smear positive than non-diabetic patients (88% vs 59%, p<0.01).[19] Singla et al reported that diabetic TB patients was more likely to have higher grade of smear positivity than non-diabetic TB patients (65.2% vs 54.1%, p = 0.008).[20] Park et al reported that there was no difference in clinical symptoms between diabetics and non-diabetics and that patients with poor diabetic control were more likely to be smear positive compared with non-diabetes.[21] Most of these articles had a relatively small sample size and were not able to take covariates into account in assessing the association between DM and clinical manifestations of TB.[22–24] Our study enrolled more than 700 diabetic pulmonary TB patients and revealed that diabetic patients were significantly more likely to have any symptom, cough, hemoptysis, tiredness weight loss, positive smear and higher smear positivity grades as compared with non-diabetics after adjusting for covariates. Furthermore, the association between diabetes and clinical manifestations of pulmonary TB was related to glycemic control. Patients with poor glycemic control had more symptoms and were more likely to be smear positive with a higher positivity grade. This suggests that proper glycemic control may reduce the frequency of symptoms and smear positivity grades, hence the risk of transmission of tuberculous infection.

### Sputum culture positive at 2–3 months

The association between DM and remaining sputum culture positive after 2–3 months of TB treatment has previously been investigated, but results were heterogeneous and inconsistent.[13] Again, sample size of diabetic patients in most studies was relatively small, with insufficient power to assess whether there was systematic difference between diabetes and non-diabetes. Jiménez-Corona et al reported that patients with DM and pulmonary TB had delayed sputum conversion (aOR 1.51, 95% CI 1.09 to 2.10). [8] Park et al reported that poorly controlled diabetes was a significant risk factor for a positive sputum culture at 2 months (odds ratio, 4.316; 95% CI, 1.306–14.267; p = 0.017).[21] Our data confirms Park’s findings that remaining sputum culture positive at 2 months was related to glycemic control.

### TB treatment outcome

A recent meta-analysis on the impact of diabetes on TB treatment outcome reported that diabetic patients have a risk ratio (RR) for the combined outcome of failure and death of 1.69 (95% CI, 1.36 to 2.12); the RR of death during TB treatment was 1.89 (95% CI, 1.52 to 2.36) among the 23 unadjusted studies, and 4.95 (95% CI, 2.69 to 9.10) among the 4 studies that adjusted for age and other potential confounding factors. [18, 25–27] Jiménez-Corona et al reported that patients with DM and pulmonary TB had a higher probability of treatment failure (aOR 2.93, 95% CI 1.18 to 7.23), recurrence (adjusted HR (aHR) 1.76, 95% CI 1.11 to 2.79) and relapse (aHR 1.83, 95% CI 1.04 to 3.23).[8] Our study confirms that DM is associated with unfavorable outcome. However, the influence of DM on outcome of TB treatment was not proportionately related to pretreatment HbA1C, a marker of short term glycemic control, but was mainly mediated through diabetes-related comorbidities, which might reflect the effect of long term inadequate glycemic control [28]. The analysis on one year mortality confirmed that diabetes-related comorbidity was one of the driving forces of mortality among diabetic TB patients. This likely implies that long term glycemic control before the development of TB will be critical in improving outcome of TB in diabetic patients.

### DM and drug-resistant TB

The association between diabetes and drug-resistant TB has been investigated previously and results reported were not consistent. Hsu et al reported that diabetes was associated with H resistance but not MDR-TB in both new and previously treated TB cases.[29] Chang et al reported that diabetic patients were more likely to develop resistance to R during treatment.[19] A recently published systematic review and meta-analysis reported that diabetes was not associated with an increased risk of recurrent disease with drug-resistant TB.[13] Our study found that diabetes was not associated with amplification of resistance to H or to R. However, our study was not powered to investigate the association between diabetes and amplification of resistance during treatment. Ruslami et al reported that diabetes does not alter the pharmacokinetics of anti-TB drugs during the intensive phase of TB treatment.[30] However, Babalik et al reported that plasma H and R concentrations were decreased by about 50% in diabetic pulmonary TB patients,[31] which may increase the risk of acquired resistance during treatment.

### Strengths and limitations

Our study has several strengths. First, we enrolled more than 700 diabetic pulmonary TB patients which enabled us to take other covariates of clinical manifestations and outcome of TB into account in analysis. Second, we not only investigated the influence of diabetes on TB but also glycemic control. The finding that the influence of diabetes on TB was related to glycemic control implies that the impact of diabetes on TB is modifiable; early diagnosis of diabetes and adequate glycemic control of the majority of diabetic patients have great potential in mitigating the impact of diabetes on TB at population level. Our study has limitations. It is a hospital-based study. TB may aggravate hyperglycemia. It was difficult to determine the causal pathway (temporal relationship) of glycemic control and clinical manifestations of TB in a retrospective cohort study that did not have information on glycemic control before the onset of TB. We were not able to refute the possibility of reverse causality that poor glycemic control (A1C>9%) was in part caused by severe TB, hence the association between poor glycemic control and a higher frequency of symptoms and a positive smear with higher smear positivity grades. However, given that poor glycemic control is likely associated with a higher risk of TB, it is unlikely that reactive hyperglycemia caused by TB is the main mechanism of our observations. Although the mechanisms by which diabetes modifies the presentation and course of TB are not yet clearly understood, studies have shown that dysfunctional innate and adaptive immune response to TB in diabetic patients is related to hyperglycemia.[32–37] Similar to other diabetic complications, the influence of diabetes on TB is related to the cumulative effect of chronic hyperglycemia [28, 36, 37] The initiation of adaptive immunity is impaired by chronic hyperglycemia, resulting in a higher bacillary burden and more extensive inflammation. [33, 37] Restrepo et al. reported that innate and type 1 cytokine responses were significantly higher in diabetic patients with TB than in non-diabetic control and the effect was consistently and significantly more marked in diabetic patients with chronic hyperglycemia. [32] Kumar et al reported that TB in diabetic patients is characterized by elevated frequencies of Th1 and Th17 cells, indicating that an alteration in the immune response to TB leading to a biased induction of Th1- and Th17-mediated cellular responses which likely contribute to increased immune pathology in *M*. *tuberculosis* infection.[34] Therefore, it is likely that proper glycemic control may reduce the impact of diabetes on TB, including risk of TB, severity of TB, and outcome of treatment.

### Future studies

To clarify whether the association between poor glycemic control and a higher frequency of symptoms and a positive smear with higher smear positivity grades observed in our study was due to reverse causality that poor glycemic control was caused by severe TB, a prospective cohort study is needed to disentangle the causal pathway of poor glycemic control and the severity of TB. To what extend the unsatisfactory outcome of TB among diabetic patients can be averted by proper glycemic control remains to be demonstrated. Further studies are also needed to clarify whether diabetes is associated with an increased risk of drug-resistant TB.
